# Supplementary material for: Inflammatory factors and risk of lung adenocarcinoma: a Mendelian randomization study mediated by blood metabolites
Source: Front Endocrinol (Lausanne). 2024 Aug 27;15:1446863. doi: 10.3389/fendo.2024.1446863 (PMC11384989; doi:10.3389/fendo.2024.1446863)
Supplement: Supplementary file 1 [file DataSheet1.zip › supplementary files/S2/GCST90200326/forest.pdf]

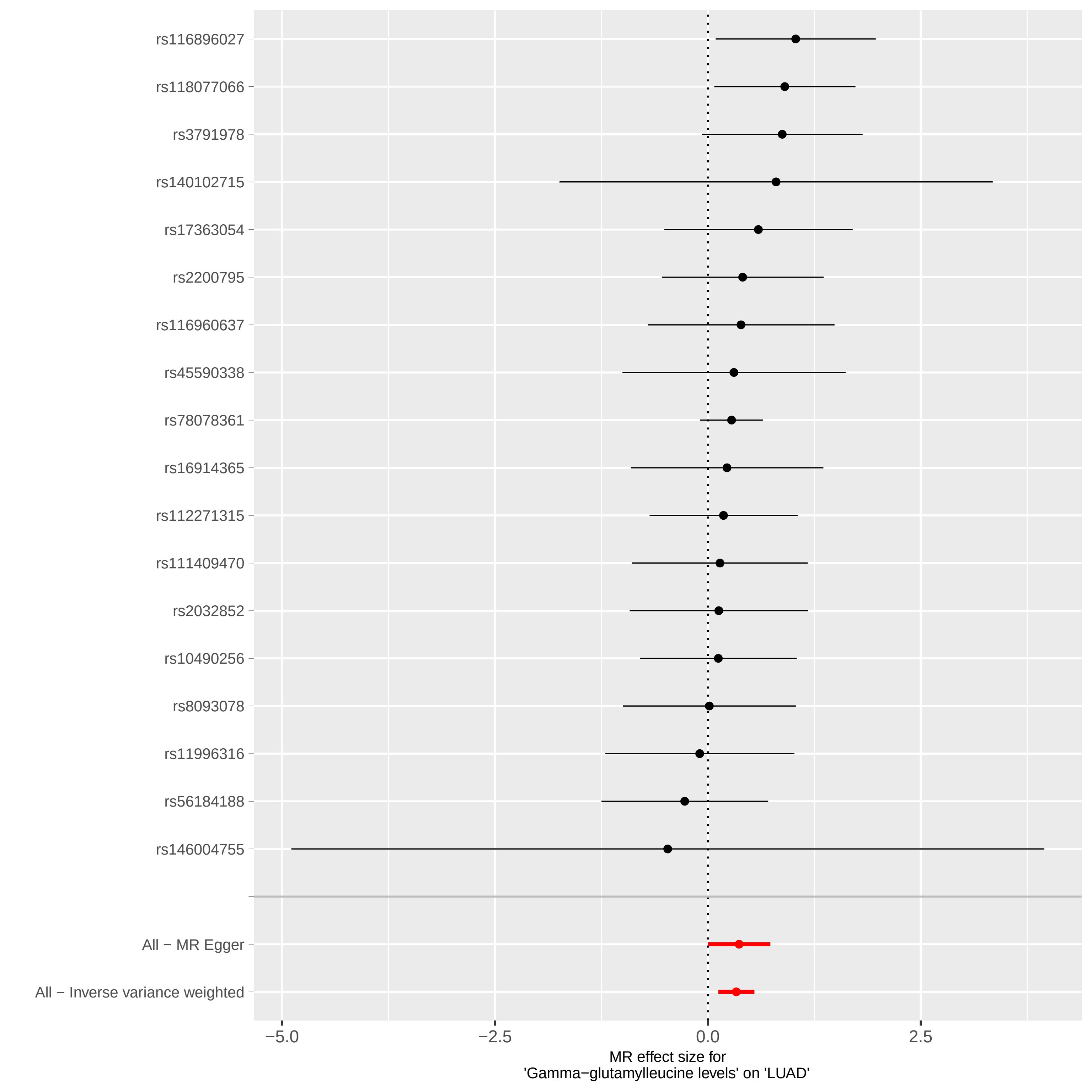

rs116896027
rs118077066
rs3791978
rs140102715
rs17363054
rs2200795
rs116960637
rs45590338
rs78078361
rs16914365
rs112271315
rs111409470
rs2032852
rs10490256
rs8093078
rs11996316
rs56184188
rs146004755
All − MR Egger
All − Inverse variance weighted
−5.0
−2.5
0.0
2.5
MR effect size for
'Gamma−glutamylleucine levels' on 'LUAD'
